# *catena*-Poly[[triphenyl­tin(IV)]-μ-2-(2-picolinoylhydrazono)propanoato-κ^2^ *O* ^1^:*O* ^2^]

**DOI:** 10.1107/S1600536809039981

**Published:** 2009-10-07

**Authors:** Jichun Cui, Yanling Qiao, Longhua Ding, Handong Yin

**Affiliations:** a College of Chemistry and Chemical Engineering, Liaocheng University, Shandong 252059, People’s Republic of China

## Abstract

In the title polymeric coordination compound, [Sn(C_6_H_5_)_3_(C_9_H_8_N_3_O_3_)]_*n*_, the Sn^IV^ atom is in a distorted trigonal-bipyramidal geometry, being coordinated by two O atoms from two 2-(2-picolinoylhydrazono)propanoate ligands and three phenyl groups. Adjacent Sn atoms are bridged by the 2-(2-picolinoylhydrazono)propanoate ligand through one carbonyl O atom and one carboxyl­ate O atom, forming a chain structure propagating parallel to [100]. An intra­molecular N—H⋯O hydrogen bond is observed.

## Related literature

For some organotin(IV) complexes with pyruvic acid iso­nicotinyl hydrazone, see: Yin *et al.* (2005 ▶).
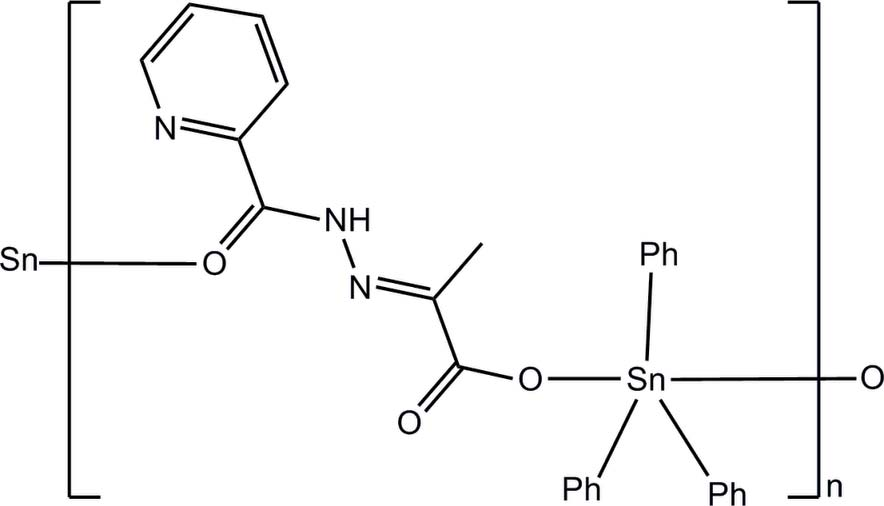


## Experimental

### 

#### Crystal data

[Sn(C_6_H_5_)_3_(C_9_H_8_N_3_O_3_)]
*M* *_r_* = 556.17
Orthorhombic, 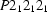

*a* = 10.2622 (9) Å
*b* = 11.0105 (12) Å
*c* = 22.344 (2) Å
*V* = 2524.6 (4) Å^3^
*Z* = 4
Mo *K*α radiation
μ = 1.04 mm^−1^
*T* = 298 K
0.33 × 0.20 × 0.15 mm

#### Data collection

Siemens SMART 1000 CCD diffractometer
Absorption correction: multi-scan (*SADABS*; Sheldrick, 1996 ▶) *T*_min_ = 0.724, *T*_max_ = 0.859
10482 measured reflections
4441 independent reflections
3863 reflections with *I* > 2σ(*I*)
*R*_int_ = 0.033

#### Refinement

*R*[*F* ^2^ > 2σ(*F* ^2^)] = 0.033
*wR*(*F* ^2^) = 0.068
*S* = 1.00
4441 reflections
308 parameters
H-atom parameters constrained
Δρ_max_ = 0.50 e Å^−3^
Δρ_min_ = −0.32 e Å^−3^
Absolute structure: Flack (1983 ▶), 1905 Friedel pairs
Flack parameter: −0.01 (2)

### 

Data collection: *SMART* (Siemens, 1996 ▶); cell refinement: *SAINT* (Siemens, 1996 ▶); data reduction: *SAINT*; program(s) used to solve structure: *SHELXS97* (Sheldrick, 2008 ▶); program(s) used to refine structure: *SHELXL97* (Sheldrick, 2008 ▶); molecular graphics: *SHELXTL* (Sheldrick, 2008 ▶); software used to prepare material for publication: *SHELXTL*.

## Supplementary Material

Crystal structure: contains datablocks I, global. DOI: 10.1107/S1600536809039981/hy2232sup1.cif

Structure factors: contains datablocks I. DOI: 10.1107/S1600536809039981/hy2232Isup2.hkl

Additional supplementary materials:  crystallographic information; 3D view; checkCIF report

## Figures and Tables

**Table 1:** Selected bond lengths (Å)

Sn1—O2^i^	2.123 (3)
Sn1—O3	2.549 (3)
Sn1—C10	2.130 (4)
Sn1—C16	2.134 (4)
Sn1—C22	2.129 (4)

Symmetry code: (i) 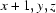.

**Table 2:** Hydrogen-bond geometry (Å, °)

*D*—H⋯*A*	*D*—H	H⋯*A*	*D*⋯*A*	*D*—H⋯*A*
N2—H2⋯O1	0.86	1.99	2.647 (5)	132

**Fig. 1.**
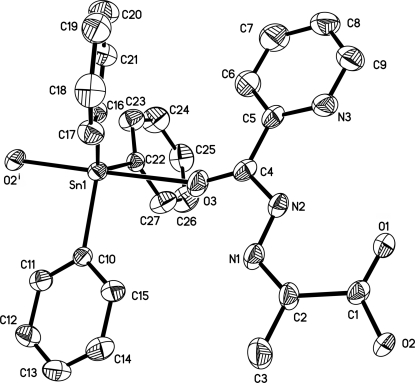
The asymmetric unit of the title compound, showing the 50% probability displacement ellipsoids. H atoms have been omitted for clarity. [Symmetry code: (i) x+1, y, z.]

## supplementary crystallographic information

### Comment

Recently, we have reported some organotin(IV) complexes with pyruvic acid isonicotinyl hydrazone (Yin *et al.*,  2005). As a part of our ongoing investigations in this field, we have synthesized the title compound and present its crystal structure here.

The title compound (Fig. 1) forms an extended one-dimensional chain structure arising from Sn—O bridges formed by the 2-(2-picolinoylhydrazono)propanoate ligand. The Sn^IV^ atom assumes a distorted trigonal-bipyramidal coordination geometry, with atoms O3 and O2^i^ in the axial positions [O3—Sn1—O2^i^ = 175.65 (13)°, symmetry code: (i) x+1, y, z] and the atoms C10, C16 and C22 in the equatorial positions. One of the two Sn—O bond lengths is shorter and the other is longer (Table 1). The complex involves an intramolecular N—H···O hydrogen bond (Table 2).

### Experimental

The reaction was carried out under nitrogen atmosphere. 2-(2-Picolinoylhydrazono)propanoic acid (1 mmol) and sodium ethoxide (1.2 mmol) were added to a solution of benzene (30 ml) in a Schlenk flask and stirred for 0.5 h. Triphenyltin chloride (1 mmol) was then added to the reactor and the reaction mixture was stirred for 4 h at 313 K. The resulting clear solution was evaporated under vacuum. The product was crystallized from a mixture of dichloromethane/methanol (v/v 1:1) to yield colorless block crystals of the title compound (yield 78%).

### Refinement

H atoms were positioned geometrically and refined as riding atoms, with C—H = 0.93 (aromatic) and 0.96 (methyl) Å, and N—H = 0.86 Å and with *U*_iso_(H) = 1.2(1.5 for methyl)*U*_eq_(C,N).

### Crystal data

**Table:**

[Sn(C_6_H_5_)_3_(C_9_H_8_N_3_O_3_)]	*F*(000) = 1120
*M**_r_* = 556.17	*D*_x_ = 1.463 Mg m^−^^3^
Orthorhombic, *P*2_1_2_1_2_1_	Mo *K*α radiation, λ = 0.71073 Å
Hall symbol: P 2ac 2ab	Cell parameters from 4196 reflections
*a* = 10.2622 (9) Å	θ = 2.6–23.1°
*b* = 11.0105 (12) Å	µ = 1.04 mm^−^^1^
*c* = 22.344 (2) Å	*T* = 298 K
*V* = 2524.6 (4) Å^3^	Block, colorless
*Z* = 4	0.33 × 0.20 × 0.15 mm

### Data collection

**Table:**

Siemens SMART 1000 CCD diffractometer	4441 independent reflections
Radiation source: fine-focus sealed tube	3863 reflections with *I* > 2σ(*I*)
graphite	*R*_int_ = 0.033
φ and ω scans	θ_max_ = 25.0°, θ_min_ = 1.8°
Absorption correction: multi-scan (*SADABS*; Sheldrick, 1996)	*h* = −12→12
*T*_min_ = 0.724, *T*_max_ = 0.859	*k* = −13→11
10482 measured reflections	*l* = −11→26

### Refinement

**Table:**

Refinement on *F*^2^	Secondary atom site location: difference Fourier map
Least-squares matrix: full	Hydrogen site location: inferred from neighbouring sites
*R*[*F*^2^ > 2σ(*F*^2^)] = 0.033	H-atom parameters constrained
*wR*(*F*^2^) = 0.068	*w* = 1/[σ^2^(*F*_o_^2^) + (0.0302*P*)^2^ + 0.4754*P*] where *P* = (*F*_o_^2^ + 2*F*_c_^2^)/3
*S* = 1.00	(Δ/σ)_max_ = 0.001
4441 reflections	Δρ_max_ = 0.50 e Å^−^^3^
308 parameters	Δρ_min_ = −0.32 e Å^−^^3^
0 restraints	Absolute structure: Flack (1983), 1905 Friedel pairs
Primary atom site location: structure-invariant direct methods	Flack parameter: −0.01 (2)

### Fractional atomic coordinates and isotropic or equivalent isotropic displacement parameters (Å^2^)

**Table:**

	*x*	*y*	*z*	*U*_iso_*/*U*_eq_	
Sn1	0.44055 (3)	0.91214 (3)	0.881010 (13)	0.04040 (9)	
N1	−0.0246 (3)	0.8712 (3)	0.86140 (18)	0.0525 (11)	
N2	−0.0091 (4)	0.9927 (4)	0.84881 (17)	0.0500 (11)	
H2	−0.0749	1.0367	0.8389	0.060*	
N3	0.0110 (4)	1.2185 (4)	0.8083 (2)	0.0679 (13)	
O1	−0.2663 (3)	1.0095 (3)	0.84524 (16)	0.0615 (10)	
O2	−0.3655 (3)	0.8450 (3)	0.88128 (16)	0.0525 (8)	
O3	0.2059 (3)	0.9861 (3)	0.87271 (16)	0.0609 (9)	
C1	−0.2661 (4)	0.9054 (5)	0.86320 (18)	0.0470 (11)	
C2	−0.1409 (4)	0.8298 (4)	0.8660 (2)	0.0536 (14)	
C3	−0.1536 (5)	0.6967 (4)	0.8767 (4)	0.095 (2)	
H3A	−0.0689	0.6597	0.8753	0.143*	
H3B	−0.2081	0.6617	0.8464	0.143*	
H3C	−0.1918	0.6831	0.9153	0.143*	
C4	0.1115 (5)	1.0416 (5)	0.8524 (2)	0.0490 (13)	
C5	0.1188 (5)	1.1707 (5)	0.8323 (2)	0.0524 (13)	
C6	0.2312 (6)	1.2354 (5)	0.8391 (3)	0.0771 (17)	
H6	0.3048	1.1992	0.8556	0.092*	
C7	0.2344 (7)	1.3558 (6)	0.8210 (3)	0.093 (2)	
H7	0.3099	1.4016	0.8256	0.111*	
C8	0.1258 (6)	1.4063 (6)	0.7964 (3)	0.0855 (18)	
H8	0.1258	1.4865	0.7833	0.103*	
C9	0.0156 (6)	1.3348 (6)	0.7914 (3)	0.084 (2)	
H9	−0.0593	1.3696	0.7755	0.101*	
C10	0.3724 (4)	0.7494 (4)	0.92196 (19)	0.0416 (11)	
C11	0.4586 (6)	0.6846 (4)	0.9577 (2)	0.0563 (13)	
H11	0.5426	0.7141	0.9633	0.068*	
C12	0.4220 (6)	0.5767 (5)	0.9851 (2)	0.0695 (14)	
H12	0.4816	0.5342	1.0084	0.083*	
C13	0.2987 (7)	0.5332 (5)	0.9778 (3)	0.0774 (18)	
H13	0.2736	0.4610	0.9960	0.093*	
C14	0.2120 (6)	0.5972 (6)	0.9432 (3)	0.0807 (17)	
H14	0.1276	0.5679	0.9383	0.097*	
C15	0.2481 (5)	0.7040 (5)	0.9156 (2)	0.0613 (15)	
H15	0.1878	0.7460	0.8923	0.074*	
C16	0.4224 (4)	0.9300 (4)	0.78627 (17)	0.0401 (10)	
C17	0.3600 (5)	0.8390 (4)	0.7544 (2)	0.0567 (13)	
H17	0.3265	0.7719	0.7745	0.068*	
C18	0.3471 (6)	0.8474 (6)	0.6925 (3)	0.0701 (16)	
H18	0.3047	0.7861	0.6715	0.084*	
C19	0.3964 (5)	0.9455 (6)	0.6623 (2)	0.0710 (17)	
H19	0.3889	0.9502	0.6209	0.085*	
C20	0.4566 (6)	1.0359 (5)	0.6933 (2)	0.0681 (15)	
H20	0.4886	1.1033	0.6730	0.082*	
C21	0.4705 (5)	1.0281 (4)	0.7553 (2)	0.0564 (13)	
H21	0.5127	1.0901	0.7759	0.068*	
C22	0.4531 (5)	1.0551 (4)	0.94492 (18)	0.0418 (10)	
C23	0.5361 (6)	1.1514 (4)	0.9415 (2)	0.0656 (15)	
H23	0.5927	1.1580	0.9091	0.079*	
C24	0.5371 (7)	1.2402 (5)	0.9860 (3)	0.0850 (19)	
H24	0.5938	1.3058	0.9826	0.102*	
C25	0.4572 (7)	1.2324 (5)	1.0339 (3)	0.0777 (17)	
H25	0.4569	1.2930	1.0629	0.093*	
C26	0.3775 (7)	1.1350 (6)	1.0388 (3)	0.085 (2)	
H26	0.3244	1.1269	1.0723	0.102*	
C27	0.3745 (5)	1.0475 (5)	0.9946 (2)	0.0698 (16)	
H27	0.3179	0.9820	0.9986	0.084*	

### Atomic displacement parameters (Å^2^)

**Table:**

	*U*^11^	*U*^22^	*U*^33^	*U*^12^	*U*^13^	*U*^23^
Sn1	0.03474 (14)	0.04058 (15)	0.04589 (15)	−0.00566 (16)	0.00029 (17)	0.00224 (16)
N1	0.032 (2)	0.054 (2)	0.071 (3)	−0.0004 (16)	−0.0010 (18)	−0.0072 (19)
N2	0.030 (2)	0.060 (3)	0.060 (2)	−0.0003 (18)	−0.0007 (18)	0.001 (2)
N3	0.049 (3)	0.084 (3)	0.070 (3)	−0.009 (2)	−0.006 (2)	0.027 (3)
O1	0.0360 (19)	0.061 (2)	0.088 (3)	0.0008 (16)	0.0023 (18)	0.016 (2)
O2	0.0291 (15)	0.0616 (18)	0.067 (2)	−0.0036 (14)	0.0045 (18)	0.008 (2)
O3	0.0346 (18)	0.068 (2)	0.080 (3)	−0.0012 (16)	−0.002 (2)	−0.004 (2)
C1	0.031 (2)	0.061 (3)	0.048 (3)	−0.008 (3)	−0.0073 (19)	0.002 (3)
C2	0.033 (3)	0.050 (3)	0.077 (4)	0.003 (2)	−0.011 (2)	−0.003 (3)
C3	0.050 (3)	0.057 (3)	0.179 (7)	0.002 (3)	−0.005 (5)	0.012 (4)
C4	0.037 (3)	0.064 (3)	0.047 (3)	−0.011 (2)	0.002 (2)	0.000 (3)
C5	0.036 (3)	0.067 (3)	0.054 (3)	−0.008 (3)	0.002 (2)	−0.002 (3)
C6	0.056 (4)	0.076 (4)	0.100 (5)	−0.008 (3)	−0.005 (3)	0.013 (4)
C7	0.071 (5)	0.091 (5)	0.117 (6)	−0.025 (4)	−0.009 (4)	0.014 (4)
C8	0.077 (4)	0.080 (4)	0.099 (5)	−0.017 (4)	0.001 (4)	0.029 (4)
C9	0.073 (5)	0.089 (5)	0.091 (5)	−0.002 (4)	−0.010 (3)	0.037 (4)
C10	0.040 (3)	0.036 (2)	0.049 (3)	−0.002 (2)	0.009 (2)	−0.003 (2)
C11	0.053 (3)	0.058 (3)	0.058 (3)	−0.002 (3)	0.005 (3)	0.009 (2)
C12	0.081 (4)	0.061 (3)	0.066 (3)	0.010 (4)	0.011 (3)	0.021 (3)
C13	0.087 (5)	0.054 (3)	0.091 (4)	−0.009 (3)	0.021 (4)	0.016 (3)
C14	0.059 (4)	0.070 (4)	0.113 (5)	−0.017 (4)	0.015 (3)	0.009 (4)
C15	0.049 (3)	0.052 (3)	0.083 (4)	−0.006 (3)	0.006 (3)	0.012 (3)
C16	0.031 (2)	0.045 (2)	0.044 (2)	0.004 (2)	−0.0019 (19)	0.000 (2)
C17	0.065 (3)	0.051 (3)	0.054 (3)	−0.002 (3)	0.001 (3)	0.001 (3)
C18	0.071 (4)	0.079 (4)	0.061 (4)	−0.002 (3)	−0.015 (3)	−0.018 (3)
C19	0.068 (4)	0.095 (5)	0.051 (3)	0.010 (3)	−0.009 (3)	0.004 (3)
C20	0.070 (4)	0.074 (4)	0.061 (3)	−0.007 (3)	−0.006 (3)	0.019 (3)
C21	0.059 (4)	0.053 (3)	0.056 (3)	−0.007 (3)	−0.007 (3)	0.007 (2)
C22	0.038 (2)	0.043 (3)	0.045 (2)	0.001 (2)	−0.002 (2)	−0.0001 (18)
C23	0.074 (4)	0.056 (3)	0.067 (3)	−0.017 (3)	0.010 (3)	−0.010 (3)
C24	0.097 (5)	0.062 (4)	0.096 (5)	−0.023 (4)	0.006 (4)	−0.024 (3)
C25	0.090 (5)	0.067 (4)	0.076 (4)	0.002 (4)	−0.011 (4)	−0.030 (3)
C26	0.097 (5)	0.102 (5)	0.055 (3)	−0.003 (4)	0.016 (3)	−0.021 (4)
C27	0.074 (4)	0.074 (4)	0.062 (3)	−0.018 (3)	0.016 (3)	−0.008 (3)

### Geometric parameters (Å, °)

**Table:**

Sn1—O2^i^	2.123 (3)	C11—C12	1.388 (7)
Sn1—O3	2.549 (3)	C11—H11	0.9300
Sn1—C10	2.130 (4)	C12—C13	1.363 (8)
Sn1—C16	2.134 (4)	C12—H12	0.9300
Sn1—C22	2.129 (4)	C13—C14	1.373 (8)
N1—C2	1.281 (6)	C13—H13	0.9300
N1—N2	1.377 (5)	C14—C15	1.380 (7)
N2—C4	1.352 (5)	C14—H14	0.9300
N2—H2	0.8600	C15—H15	0.9300
N3—C9	1.336 (7)	C16—C21	1.375 (6)
N3—C5	1.337 (6)	C16—C17	1.387 (6)
O1—C1	1.215 (6)	C17—C18	1.393 (7)
O2—C1	1.283 (5)	C17—H17	0.9300
O2—Sn1^ii^	2.123 (3)	C18—C19	1.370 (8)
O3—C4	1.232 (5)	C18—H18	0.9300
C1—C2	1.532 (6)	C19—C20	1.360 (7)
C2—C3	1.490 (7)	C19—H19	0.9300
C3—H3A	0.9600	C20—C21	1.395 (6)
C3—H3B	0.9600	C20—H20	0.9300
C3—H3C	0.9600	C21—H21	0.9300
C4—C5	1.492 (7)	C22—C23	1.363 (6)
C5—C6	1.365 (7)	C22—C27	1.376 (6)
C6—C7	1.387 (8)	C23—C24	1.394 (7)
C6—H6	0.9300	C23—H23	0.9300
C7—C8	1.360 (8)	C24—C25	1.351 (8)
C7—H7	0.9300	C24—H24	0.9300
C8—C9	1.382 (8)	C25—C26	1.353 (8)
C8—H8	0.9300	C25—H25	0.9300
C9—H9	0.9300	C26—C27	1.380 (7)
C10—C15	1.378 (7)	C26—H26	0.9300
C10—C11	1.388 (6)	C27—H27	0.9300
			
O2^i^—Sn1—C22	101.46 (16)	C12—C11—H11	119.3
O2^i^—Sn1—C10	90.77 (14)	C10—C11—H11	119.3
C22—Sn1—C10	110.71 (16)	C13—C12—C11	119.9 (5)
O2^i^—Sn1—C16	96.70 (14)	C13—C12—H12	120.1
C22—Sn1—C16	127.04 (15)	C11—C12—H12	120.1
C10—Sn1—C16	118.37 (16)	C12—C13—C14	119.3 (5)
O2^i^—Sn1—O3	175.65 (13)	C12—C13—H13	120.3
C22—Sn1—O3	82.52 (15)	C14—C13—H13	120.3
C10—Sn1—O3	89.43 (14)	C13—C14—C15	121.0 (6)
C16—Sn1—O3	79.40 (13)	C13—C14—H14	119.5
C2—N1—N2	118.0 (4)	C15—C14—H14	119.5
C4—N2—N1	118.7 (4)	C10—C15—C14	120.7 (5)
C4—N2—H2	120.6	C10—C15—H15	119.6
N1—N2—H2	120.6	C14—C15—H15	119.6
C9—N3—C5	117.5 (5)	C21—C16—C17	118.4 (4)
C1—O2—Sn1^ii^	124.3 (3)	C21—C16—Sn1	122.8 (3)
C4—O3—Sn1	158.1 (3)	C17—C16—Sn1	118.9 (3)
O1—C1—O2	126.3 (4)	C16—C17—C18	120.4 (5)
O1—C1—C2	121.8 (4)	C16—C17—H17	119.8
O2—C1—C2	111.9 (4)	C18—C17—H17	119.8
N1—C2—C3	116.3 (4)	C19—C18—C17	120.4 (5)
N1—C2—C1	125.8 (4)	C19—C18—H18	119.8
C3—C2—C1	117.9 (4)	C17—C18—H18	119.8
C2—C3—H3A	109.5	C20—C19—C18	119.7 (5)
C2—C3—H3B	109.5	C20—C19—H19	120.2
H3A—C3—H3B	109.5	C18—C19—H19	120.2
C2—C3—H3C	109.5	C19—C20—C21	120.4 (5)
H3A—C3—H3C	109.5	C19—C20—H20	119.8
H3B—C3—H3C	109.5	C21—C20—H20	119.8
O3—C4—N2	123.0 (5)	C16—C21—C20	120.8 (5)
O3—C4—C5	122.9 (4)	C16—C21—H21	119.6
N2—C4—C5	114.0 (5)	C20—C21—H21	119.6
N3—C5—C6	122.6 (5)	C23—C22—C27	117.3 (4)
N3—C5—C4	117.0 (5)	C23—C22—Sn1	125.1 (3)
C6—C5—C4	120.4 (5)	C27—C22—Sn1	117.5 (3)
C5—C6—C7	119.1 (6)	C22—C23—C24	120.7 (5)
C5—C6—H6	120.4	C22—C23—H23	119.7
C7—C6—H6	120.4	C24—C23—H23	119.7
C8—C7—C6	119.3 (6)	C25—C24—C23	121.1 (6)
C8—C7—H7	120.4	C25—C24—H24	119.5
C6—C7—H7	120.4	C23—C24—H24	119.5
C7—C8—C9	118.0 (6)	C24—C25—C26	118.8 (5)
C7—C8—H8	121.0	C24—C25—H25	120.6
C9—C8—H8	121.0	C26—C25—H25	120.6
N3—C9—C8	123.5 (6)	C25—C26—C27	120.6 (6)
N3—C9—H9	118.2	C25—C26—H26	119.7
C8—C9—H9	118.2	C27—C26—H26	119.7
C15—C10—C11	117.6 (5)	C22—C27—C26	121.5 (5)
C15—C10—Sn1	124.4 (4)	C22—C27—H27	119.3
C11—C10—Sn1	118.1 (3)	C26—C27—H27	119.3
C12—C11—C10	121.4 (5)		

Symmetry codes: (i) *x*+1, *y*, *z*; (ii) *x*−1, *y*, *z*.

### Hydrogen-bond geometry (Å, °)

**Table:**

*D*—H···*A*	*D*—H	H···*A*	*D*···*A*	*D*—H···*A*
N2—H2···O1	0.86	1.99	2.647 (5)	132
